# Acute effect of Melon Manis Terengganu peel powder on glycemic response, perceived satiety, and food intake: a randomized, placebo-controlled crossover trial in adults at risk of type 2 diabetes

**DOI:** 10.1186/s40795-022-00572-1

**Published:** 2022-08-08

**Authors:** Ying Qian Ong, Sakinah Harith, Mohd Razif Shahril, Norshazila Shahidan, Hermizi Hapidin

**Affiliations:** 1 Faculty of Health Sciences, Universiti Sultan Zainal Abidin, Gong Badak Campus, 21300 Kuala Nerus, Terengganu Malaysia; 2 Nutrition Program, Center for Healthy Ageing and Wellness, Faculty of Health Sciences, Universiti Kebangsaan Malaysia, Jalan Raja Muda Abdul Aziz, 50300 Kuala Lumpur, Malaysia; 3 Faculty of Bioresources and Food Industry, Universiti Sultan Zainal Abidin, Tembila Campus, 22200 Besut, Terengganu Malaysia; 4 Biomedicine Programme, School of Health Sciences, Health Campus, Universiti Sains Malaysia, 16150 Kubang Kerian, Kelantan Malaysia

**Keywords:** Melon Manis Terengganu peel, Glycemic response, Perceived satiety, Food intake, At risk of type 2 diabetes

## Abstract

**Background:**

Melon Manis Terengganu (MMT) peel has a high dietary fiber content, but there is no data examining its health benefits in adults at risk of type 2 diabetes. The objective of the study was to evaluate whether consumption of MMT peel powder improves glycemic response, satiety, and food intake in adults at risk of type 2 diabetes.

**Methods:**

An open-label, randomized, placebo-controlled, crossover design trial was conducted among adults (*n* = 30, ages 18–59 y) at risk of type 2 diabetes. They consumed Formulation 3 (formulated MMT peel powder) [A] and control (glucose) [B] with study breakfast based on randomly assigned treatment sequences (AB, BA) established by Research Randomizer (www.randomizer.org). Capillary blood glucose and perceived satiety were determined at baseline (0 min), 30, 60, 90 and 120 min, followed by a post-intervention food intake measurement.

**Results:**

The repeated measures analysis of variance (ANOVA) revealed significant time (F = 84.37, *p* <  0.001, η_p_^2^ = 0.744), condition (F = 22.89, *p* <  0.001, η_p_^2^ = 0.441), and time*condition effects (F = 24.40, *p* <  0.001, η_p_^2^ = 0.457) in blood glucose levels. Respondents (*n* = 30) who consumed Formulation 3 also had a significantly lower blood glucose 2-hour incremental area under the curve (iAUC) of 134.65 ± 44.51 mmol/L*min and maximum concentration (CMax) of 7.20 (7.10, 8.20) mmol/L with relative reduction of 26.8 and 13.3% respectively, when compared with control (*p* <  0.001). Besides, significantly greater perceived satiety, lower energy and fat intake as well as higher dietary fiber intake were also observed in the intervention group compared with the placebo group (*p* <  0.05). There were no marked side effects associated with the ingestion of the test products.

**Conclusions:**

Short-term consumption of formulated MMT peel powder may improve glycemic response, increase perceived satiety and reduce food intake in adults at risk of type 2 diabetes with the potential to be utilized as a functional beverage. Medium-to long-term clinical trial is warranted to determine whether taking this formulated MMT peel powder on a daily basis has an influence on health outcomes.

**Trial registration:**

ClinicalTrials.gov Identifier: NCT05298111. Registered 28/03/2022.

## Background

Diabetes is a difficult challenge in the twenty-first century due to its increasing prevalence [1]. The number of adults diagnosed with diabetes has risen substantially from 108 million in 1980 to 537 million in 2021, with prediction that this number would increase to 643 million by 2030 and 783 million by 2045 [2, 3]. Surprisingly, 541 million people have impaired glucose tolerance worldwide [3]. In Malaysia, one in five adults has diabetes which translates to about 3.9 million people aged 18 years and above. The diabetes prevalence has increased from 11.2% in 2011 to 18.3% in 2019 [4]. The increasing trend indicates the need for the implementation of preventive approaches.

Dietary interventions are still fundamental and one of the most effective and safest strategies to control glucose levels [5, 6]. Large epidemiological studies propose that dietary fiber is one of the most effective nutrient components for diabetes prevention [7]. Melon Manis Terengganu (MMT) peel is a food source with high dietary fiber of 49.25 (2.05) g/100 g [8]. MMT belongs to the Cucurbitaceae family, which is a member of the *Cucumis melo* L*.* species. It is also known as *Cucumis melo* var. Inodorus cv. Manis Terengganu 1 [9]. It consisted of 28–30% of peel [10]. Existing literature demonstrated that *Cucumis melo* L. peel consisted of bioactive compounds, vitamins and minerals with beneficial health benefits [10, 11].

To date, far too little attention has been paid to the utilization of MMT peel, which is discarded as waste. Also, there is no human clinical trial exploring the beneficial health effects of MMT peel powder consumption in standardized doses or reasonable amounts among populations at risk of type 2 diabetes. Hence, this study was necessary in order to fill the gaps addressed. It is hoped that this study will make important contributions to the field of diabetes mellitus since the preliminary data on the role of MMT peel as a glycemic control agent can be established. Hence, the purpose of this study was to investigate the acute effect of formulated MMT peel powder consumption on glycemic response, perceived satiety, and food intake in adults at risk of type 2 diabetes.

## Methods

### Study design

An open label, crossover, placebo-controlled randomized controlled trial design involved two different test products namely control (glucose) and Formulation 3 (formulated MMT peel powder) was conducted in Kuala Nerus, Terengganu, Malaysia. The respondents consumed Formulation 3 (A) and control (B) with study breakfast based on randomly assigned treatment sequences (AB, BA). The randomization plan was established by Research Randomizer (www.randomizer.org) prior to the start of the study. Respondents were assigned to the randomization plan in order of recruitment. The principal investigator generated the randomization plan, enrolled and assigned respondents to the interventions. Each study visit was separated into three days of washout period and lasted about 3 hr. (starting around 8 am and finishing at approximately 11 am). There should be at least of a 3-day gap between the test meals to minimize any carryover effect [12, 13]. The study recruitment and screening started from 18th August 2021 to 18th October 2021 whereas study visits commenced from 1st October 2021 to 3rd November 2021.

### Subjects

Men and women between the ages of 18 and 59 who live, work, or study on Kuala Nerus, Terengganu, Malaysia were recruited using poster disseminated via email, social media platforms such as Facebook and Whatsapp as well as through direct approach at each department in Gong Badak Campus, Universiti Sultan Zainal Abidin (UniSZA). The respondent eligibility was identified by a two-stage screening process. In the first stage, the respondents who are at risk of type 2 diabetes mellitus (T2DM) were assessed via the modified Finnish Type 2 Diabetes Risk Assessment Tool (FINDRISC) [14] and a list of screening questions (inclusion and exclusion criteria) which were distributed via Google Form. Inclusion criteria included respondents who were Malaysian and non-smoker. On the other hand, respondents were excluded with a clinical history of T2DM; capillary fasting plasma glucose (FPG) ≥ 7.0 mmol/L; taking oral antidiabetic agents; participating in other weight management programmes or interventional research; on a prescribed medical diet; gastrointestinal tract (GIT) illnesses or conditions; allergy or sensitivity to study products; being pregnant, currently breastfeeding, or planning to become pregnant; on dietary restrictions and smokers. Those who scored ≥4 in FINDRISC (moderate or high risk for T2DM for the next 10 years) and met the inclusion criteria were invited to the second stage screening test via the capillary FPG measurement by finger pricking. In this stage, the respondents attending the preliminary visit with FPG < 7.0 mmol/L were invited to volunteer.

### Preliminary visit

During the preliminary visit, the study objective, procedure involved, expected study timeframe, respondent’s roles and other UniSZA Human Research Ethics Committee (UHREC) requirements were explained to the respondents. They were allowed to ask questions and were given ample time to examine their options. The respondents were volunteered to join this study and they can withdraw from the study as they wished. All eligible respondents were required to sign an informed consent form before participating in this study. The socio-demographic questionnaire, 24-hr dietary recalls (food logs) and International Physical Activity Questionnaire-Short Form (IPAQ-SF) were given to respondents to complete at their preliminary visit. These questionnaires were provided in both open-ended and closed-ended formats.

### Test products

The control was made up of glucose whereas Formulation 3 made up of 40% MMT peel (13.7 g) and 60% monk fruit sweetener (20.5 g). The composition of the MMT peel was displayed in Table 1. Carboxymethyl cellulose, citric acid and orange flavoring were added to the formulations and mixed thoroughly for uniform distribution of ingredients [15]. The MMT was harvested at UniSZA Agropreneur Park, Besut Campus, Terengganu. MMT was first peeled and the peel was then processed into powder, which was stored in an airtight container in a freezer (− 21 °C) prior to analyses [16]. The amount of Formulation 3 used was based on the dietary fiber content of 5 g as reported in an acute intervention study using orange pomace test beverages which contain 5.48 g of dietary fiber [17]. The weight for each product were 4.5 g of control and 36.0 g of Formulation 3. The control was given in the placebo group while Formulation 3 was provided in the intervention group.

**Table 1:** Nutritional compositions of MMT peel

Nutrients	Amount (g/100 g)
Calories (kcal)	337.23 ± 1.63
Total Carbohydrate	67.65 ± 0.36
Dietary Fiber	49.25 ± 2.05
Protein	13.31 ± 0.05
Fat	1.49 ± 0.35

The nutritional compositions of control and Formulation 3 containing similar available carbohydrate (4.5 g) are presented in Table 2. The carbohydrate content of Formulation 3 comprised of 20.5 g of sweetener with 0.02 kcal/g. This erythritol sweetener originates from the family of sugar alcohol also known as polyol, which is neither dietary fiber nor available carbohydrate. It cannot be metabolized by the human body and is excreted unmodified into urine without influencing blood glucose and insulin levels [18]. Hence, the available carbohydrate was calculated using the formula: total carbohydrate content (30 g) – sweetener content (20.5 g) - dietary fiber content (5 g) [19]. Available carbohydrates refers to the carbohydrates that are digested and absorbed by the human small intestine which can raised blood glucose levels meanwhile dietary fiber refers to carbohydrate polymers (such as lignin, cellulose, hemicellulose, gums, pectin and inulin) with 10 or more monomeric units which cannot be degraded by the endogenous digestive enzyme in the human small intestine [20–22]. On study visits, a total volume of 380 mL was consumed to maintain a standardized volume of drink between control and Formulation 3 study visits.

**Table 2:** Nutritional compositions between control and Formulation 3

Nutrients	Control	Formulation 3
Weight (g)	4.5	36.0
Calories (kcal)	18	36
Total Carbohydrates (g)	4.5	30.0
Available Carbohydrate (g)	4.5	4.5
Dietary Fiber (g)	0.0	5.0
Protein (g)	0.0	1.4
Fat (g)	0.0	0.2

The breakfast used in this study is typical foods consumed by Malaysian. The standardized breakfast consisted of 2 slices of white bread (brand Gardenia), 1 piece of hard-boiled egg, 1 teaspoon of unsalted margarine (brand Anchor) and 1 glass of plain water (200 mL), packing up to a total of 267 kcal (30.6 g carbohydrate, 11.1 g protein, 10.7 g fat and 0.9 g dietary fiber). The study meal for breakfast (test products and standardized breakfast) of both placebo and intervention groups were provided in Table 3. The total calories content of the study breakfast adhered closely to the recommendation of 400 kcal [23].

**Table 3:** Nutritional compositions of study meal (test products and standardized breakfast)

Nutrients	Placebo	Intervention
Calories (kcal)	285	303
Total Carbohydrates (g)	35.1	60.6
Available Carbohydrate (g)	35.1	35.1
Dietary Fiber (g)	0.9	5.9
Protein (g)	11.1	12.5
Fat (g)	10.7	10.9

### Data collection

The respondents attended the lab at 8 am with light clothing and were asked a series of questions to measure fasting, nutrition, physical activity and sleep regimen adherence. They were also required to hand in the completed questionnaire which consisted of dietary and physical activity information which were distributed to them prior to data collection. Then, height, weight, waist circumference (WC), % body fat and blood pressure measurement were taken followed by a perceived satiety measurement and fasting capillary plasma sample (baseline) withdrawal using finger pricking method.

Next, the respondents were randomized to consume of one of the two types of test products namely A: control and B: Formulation 3 dissolved in 180 mL plain water together with the standardized breakfast in treatment sequences (AB, BA). Each respondent consumed the meals in a separate place and avoided using electronic gadgets while eating to reduce distractions. They were instructed to consume the meals at a comfortable pace within 15 min. The respondents were asked to remain rested in the same place during the testing period and allowed to read, use their phone or laptop and use the toilet after meals consumption.

At the first bite, a timer was started and postprandial blood samples as well as perceived satiety measurement were then taken at 30, 60, 90 and 120 min thereafter. The blood collection techniques were adhered exactly as they were written in the standard operating procedure (SOP). After the 120 min assessments, the respondents were allowed to leave the testing site and instructed to record the food consumed for the rest of the day. The food record was aimed to explore the changes in food intake following each test food relative to usual food intake [24]. Also, they were given a new food log form and physical activity diary to fill in one day before the next study visit.

### Blood glucose level measurements

FPG and postprandial blood glucose were measured using glucometer (Accu Chek Performa, New South Wales, Australia) from finger-prick blood samples according to the SOP [25]. The respondents’ finger was cleaned with an alcohol swab, left to dry and pricked with a sterile lancet to obtain a drop of blood which was placed onto the glucose meter strip. The digital result was displayed in the display window after 10 to 20 seconds and then recorded. Glucometer is a fast and valid device to evaluate blood glucose level [26]. Calibration was performed using a standard glucose strip provided by the manufacturer. In comparison to venous blood glucose, capillary blood glucose is recommended due to its simplicity to obtain, greater rise in blood glucose and less variability of the results [27].

### Perceived satiety

Perceived satiety measurements were rated using a 10-cm visual analogue scale which consisted of hunger (How hungry are you?), fullness (How full are you?), desire to eat (How strong is your desire to eat?), prospective food consumption (How much would you be able to eat right now?) and thirstiness (How thirsty are you?) [28]. Respondents were asked to make a vertical mark across the line corresponding to their feelings at the present time from not at all to extremely.

### Food intake

The respondents were given a detailed verbal and written instructions as well as visual aids including image examples by the researcher on how to fill in the food log. The food and beverage consumed was recorded in the form of standard household measurements such as scoop, cup, bowl, teaspoon, dessert spoon or tablespoon. These household measures were shown to the respondents to enhance the quality of the outcomes. This can facilitate the respondents in distinguishing the amount of portion size taken relative to a given portion size. The respondents were asked to record all the food or drink they had consumed including sauces, soup and gravies once they woke up in the morning until they went to sleep at night. They were also required to provide precise information such as food preparation methods, the inclusion of condiments or spices, household quantity, products’ brand or snacking throughout the day. For respondents who ate a mixed dish such as fried rice, fried noodle and noodle soup, all the ingredients available in the mixed dish were listed down and estimated the amount in household measurements.

All the units in household measurement were converted to grams by using Malaysian Food Composition table and Malaysian Atlas of Food Exchanges and Portion Size. The macronutrients and chosen micronutrients were determined according to the Nutrient Composition of Malaysian Foods Database (https://myfcd.moh.gov.my/) and Malaysian Atlasof Food Exchanges and Portion Size. However, this database did not provide complete information on dietary fiber for all food items. Therefore, the food database from United States Department of Agriculture Food Composition Databases (https://ndb.nal.usda.gov/ndb/), Energy and Nutrient Composition of Foods Singapore (https://focos.hpb.gov.sg/eservices/ENCF/) and nutrition facts on the food packaging were used to complement the incompleteness of the Malaysian Food Composition Database.

### Statistical analysis

The data were analyzed using IBM SPSS system version 21.0 and Nutritionist Pro™ software (Version 7.0.0, Axxya Systems). The Shapiro-Wilk test was done for the assessment of normality tests and the data considered to be normal if *p* > 0.05. The 2 hr. area under the curve (AUC) of blood glucose levels (incremental area above the baseline value) and perceived satiety (hunger, fullness, desire to eat, prospective food consumption and thirstiness) were calculated using the trapezoidal approximation [29]. The maximum concentration (Cmax) of blood glucose levels were taken to be the highest concentration within the respective time interval [30].

A paired t-test (normally distributed) or Wilcoxon matched pair signed-rank sum test (non-normally distributed) was used to compare the difference of two data between groups (placebo and intervention) on blood glucose levels (iAUC and CMAx), dietary intake, and perceived satiety (AUC). Repeated measures analysis of variance (ANOVA) was used to compare the postprandial blood glucose levels between two test products over different time points, controlling for condition and time to test for condition by time interaction. The analysis was considered significant at a *p*-value ≤0.05.

The sample size for this study is based on a power calculation for CMax. A sample size of 23 respondents was determined to detect approximately a 0.62 mmol/L difference in CMax (a primary outcome measure) [30] with 80% power and at a 5% significance level. A total of 30 respondents were recruited to allow for a 20% dropout.

## Results

### Respondents’ recruitment

**Fig. 1:**
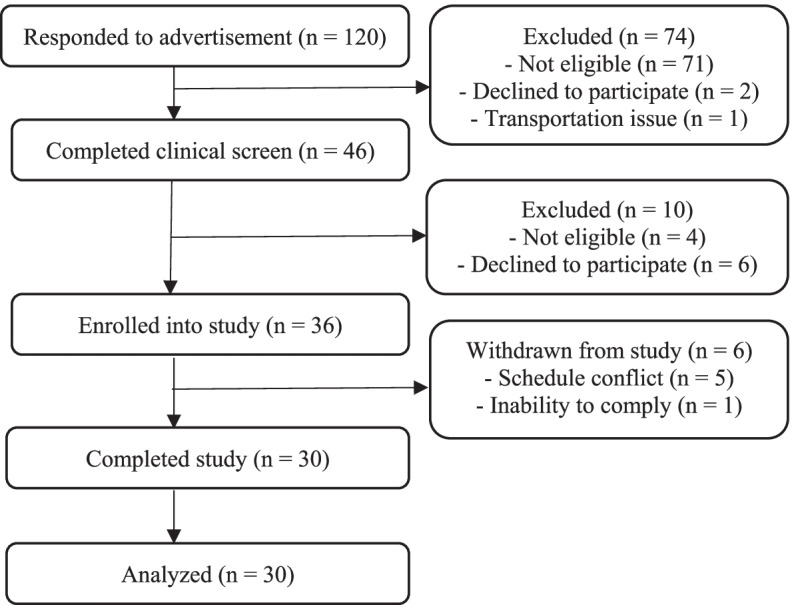
CONSORT flow diagram of the study. CONSORT, Consolidated Standards of Reporting Trials

A total of 120 Kuala Nerus locals indicated an interest in participating in the clinical experiment (Fig. 1). Out of a total of 120 individuals, 74 individuals did not match the inclusion criteria or did not start further contact. Common causes for ineligibility were FINDRISC score < 4, current breastfeeding, smoking and being diagnosed with T2DM. A total of 46 individuals enrolled during the second screening phase (capillary FPG test). Of the 46 individuals, 6 refused to engage any further due to schedule constraints while 4 had capillary FPG of > 7.0 mmol/L. On the other hand, 6 individuals were dropped from the study shortly after it began owing to non-compliance and schedule conflict. Finally, 30 respondents (7 men and 23 women) were successfully completed all two test days with 100% response rate.

### Glycemic response

The repeated measures ANOVA revealed significant time (F = 84.37, *p* <  0.001, η_p_^2^ = 0.744), condition (F = 22.89, *p* < 0.001, η_p_^2^ = 0.441), and time*condition effects (F = 24.40, *p* < 0.001, η_p_^2^ = 0.457) in blood glucose levels. There were 74.4% and 44.1% of variation associated with the time point and condition respectively whereas the time point combined with the condition types accounts for 45.7% of the variation.

Table 4 showed that there were significant differences in all comparisons in the intervention group except 30 vs 90 min (mean difference = − 0.14, 95% CI: − 0.49, 0.21; *p* = 0.429) and 30 vs 120 min (mean difference = 0.20, 95% CI: − 0.12, 0.52, *p* = 0.217). Meanwhile, all comparisons were significantly different in the placebo group. On the other hand, there was a significant difference in mean blood glucose levels among the two groups (mean difference = − 0.32, 95% CI: − 0.45, − 0.18; *p* < 0.001) regardless of time (Table 5).

**Table 4:** Comparison of blood glucose levels within each group based on time (*n* = 30)

Comparison (min)	Intervention		Placebo	
	Mean difference (95% CI)	*p*-value	Mean difference (95% CI)	*p*-value
0–30	− 1.05 (− 1.22, − 0.89)	< 0.001	− 2.05 (− 2.30, − 1.80)	< 0.001
0–60	− 1.76 (− 2.03, − 1.48)	< 0.001	− 2.51 (− 2.89, − 2.13)	< 0.001
0–90	− 1.19 (− 1.46, − 0.92)	< 0.001	− 1.31 (− 1.65, − 0.97)	< 0.001
0–120	− 0.86 (− 1.09, −-0.62)	< 0.001	− 0.43 (− 0.69, − 0.18)	0.002
30–60	−0.70 (− 1.01,-0.40)	< 0.001	−0.46 (− 0.86, − 0.06)	0.027
30–90	−0.14 (− 0.49, 0.21)	0.429	0.74 (0.35, 1.13)	0.001
30–120	0.20 (−0.12, 0.52)	0.217	1.62 (1.30, 1.94)	< 0.001
60–90	0.57 (0.28, 0.85)	< 0.001	1.20 (0.90, 1.51)	< 0.001
60–120	0.90 (0.60, 1.20)	< 0.001	2.08 (1.67, 2.49)	< 0.001
90–120	0.33 (0.11, 0.55)	0.004	0.88 (0.59, 1.17)	< 0.001

Repeated measures ANOVA within group analysis was applied followed by pairwise comparison with confidence interval adjustment by Bonferroni correction

**Table 5:** Mean difference of blood glucose levels among two groups (*n* = 30)

Comparison	MD (95% CI)	*p*-value
Intervention-placebo	−0.32 (−0.45, − 0.18)	< 0.001

Repeated measures ANOVA between group analysis was applied

Table 6 depicts significantly lower mean blood glucose levels at 30 and 60 min in the intervention group than those in the placebo group (*p* < 0.001). However, significantly higher blood glucose levels at 120 min were observed in the intervention group compared to its counterpart (*p* = 0.001). No significant difference was observed at 0 and 90 min between the two groups.

**Table 6:** Comparison of blood glucose levels among two groups based on time (*n* = 30)

Time (min)	Treatment group	Blood glucose levels (mg/mL)	95% CI	*p*-value
0	Intervention	5.63	5.47, 5.80	0.675
	Placebo	5.66	5.51, 5.81	
30	Intervention	6.69	6.45 6.92	< 0.001
	Placebo	7.71	7.46, 7.96	
60	Intervention	7.39	7.03, 7.75	< 0.001
	Placebo	8.17	7.73, 8.61	
90	Intervention	6.82	6.53, 7.12	0.280
	Placebo	6.97	6.59, 7.35	
120	Intervention	6.49	6.26, 6.72	0.001
	Placebo	6.09	5.87, 6.31	

Repeated measures ANOVA between group analysis with regard to time was applied

The blood glucose 2 hr. iAUC and Cmax of both placebo and intervention groups were summarized in Table 7. The table depicts a significantly lower blood glucose 2 hr. iAUC and Cmax in the intervention group with relative reduction of 26.8% and 13.3% respectively versus the placebo group (t(29) = 5.570, *p* < 0.001 and Z = −4.144, *p* < 0.001, respectively). In short, the results implied that the glycemic responses of the two conditions differed significantly in which intervention group demonstrated a better glycemic response.

**Table 7:** Postprandial blood glucose 2 hr. iAUC and Cmax (*n* = 30)

Type of groups	Blood glucose	
	2 hr. iAUC (mmol/L*min)	Cmax (mmol/L)
Placebo	184.05 ± 64.03	8.30 (7.90, 8.90)
Intervention	134.65 ± 44.51	7.20 (7.10, 8.20)
*p*-value	< 0.001	< 0.001

The presented results are indicated as means values ± standard deviation – 2 hr. iAUC and median values (interquartile range) - Cmax

Tested using paired t-test and Wilcoxon signed ranks test respectively

### Perceived satiety and food intake

Table 8 shows an overview of perceived satiety of both the placebo and intervention groups namely hunger, fullness, desire to eat, prospective food consumption and thirstiness from 0 to 120 min. No significant difference between groups as tested using Wilcoxon signed-rank test was observed for all parameters at 0 min (*p* > 0.05). From 60 to 120 min, the respondents in the placebo group reported a significantly greater hunger (Z = − 2.432, *p* = 0.015; Z = − 3.768, *p* < 0.001; Z = − 4.215, *p* < 0.001, respectively), desire to eat (Z = − 2.940, *p* = 0.003; Z = − 4.288, *p* < 0.001; Z = − 4.345, *p* < 0.001, respectively),and prospective food consumption (Z = − 2.961, *p* = 0.003; Z = − 4.008, *p* < 0.001; Z = − 4.201, *p* < 0.001, respectively) rating as well as lower fullness rating (Z = − 3.973, *p* < 0.001; Z = − 4.229, *p* < 0.001; Z = − 4.375, *p* < 0.001, respectively) than intervention group. For time point at 30 min, a significant difference observed in hunger (Z = − 2.849, *p* = 0.004), fullness (Z = − 2.905, *p* = 0.004) and desire to eat (Z = − 2.666, *p* = 0.008) parameter. Thirstiness was not significantly different between groups at each time point (*p* > 0.05) except at 120 min (*p* = 0.032). Perceived hunger, desire to eat, prospective food consumption and thirstiness ratings decreased postprandially at 30 min and then increased gradually from 30 min to 120 min for both treatment groups. On the other hand, perceived fullness ratings increased postprandially at 30 min and then decreased gradually from 30 min to 120 min.

**Table 8:** Perceived satiety over different time points (*n* = 30)

Time (min)	0	30	60	90	120
Hunger (cm)					
Placebo	5.4 (4.8, 6.7)	0.0 (0.0, 1.5)	1.0 (0.0, 2.6)	2.9 (0.0, 4.5)	4.3 (1.9, 6.0)
Intervention	6.0 (5.0, 7.4)	0.0 (0.0, 0.8)	0.0 (0.0, 2.1)	1.0 (0.0, 2.6)	2.3 (0.0, 4.3)
*p*-value^1^	0.162	0.004	0.015	< 0.001	< 0.001
Fullness (cm)					
Placebo	2.9 (1.2, 5.0)	10.0 (6.9, 10.0)	8.3 (6.1, 9.4)	7.1 (5.0, 8.6)	6.1 (5.0, 7.5)
Intervention	2.9 (1.2, 5.0)	10.0 (8.3, 10.0)	9.2 (8.0, 10.0)	8.4 (6.6, 9.4)	8.0 (6.3, 8.9)
*p*-value^1^	0.889	0.004	< 0.001	< 0.001	< 0.001
Desire to eat (cm)					
Placebo	5.2 (5.0, 7.1)	0.0 (0.0, 3.1)	2.0 (0.0, 3.6)	3.3 (0.9, 4.6)	5.0 (2.3, 5.9)
Intervention	6.2 (5.0, 7.2)	0.0 (0.0, 1.2)	1.1 (0.0, 2.4)	1.9 (0.0, 3.2)	2.6 (1.0, 4.1)
*p*-value^1^	0.966	0.008	0.003	< 0.001	< 0.001
Prospective food consumption (cm)					
Placebo	5.0 (3.8, 7.1)	0.0 (0.0, 3.0)	2.0 (0.0, 3.9)	4.0 (1.7, 5.0)	4.8 (2.4, 6.4)
Intervention	5.6 (4.8, 7.0)	0.0 (0.0, 2.1)	1.2 (0.0, 2.4)	2.0 (0.0, 2.9)	2.9 (1.0, 4.1)
*p*-value^1^	0.110	0.182	0.003	< 0.001	< 0.001
Thirstiness (cm)					
Placebo	5.2 (3.9, 7.0)	2.1 (0.0, 4.4)	3.0 (2.0, 6.0)	4.8 (3.1, 6.5)	7.0 (4.4, 8.4)
Intervention	5.6 (2.3, 8.9)	1.0 (0.0, 5.0)	3.0 (2.0, 6.0)	4.3 (2.1, 6.1)	5.0 (3.2, 8.0)
*p*-value^1^	0.115	0.586	1.000	0.068	0.032

The presented results are indicated as median values (interquartile range)

^1^Difference between intervention and placebo groups tested using Wilcoxon signed ranks test

**Fig. 2:**
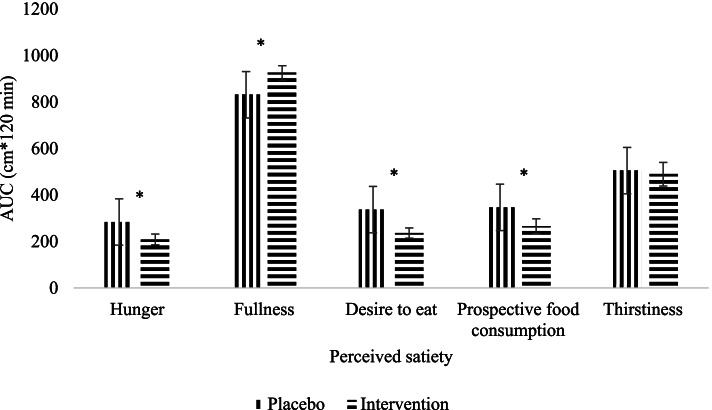
AUC of perceived satiety of placebo and intervention group. The presented results are indicated as mean values ± standard error of mean. *Significant differences between groups. Tested using paired t-test

In terms of AUC as displayed in Fig. 2, all perceived satiety parameters were significantly different between the treatment groups (*p* < 0.001) except for thirstiness (t(29) = 0.565, *p* = 0.576) in which the respondents in the intervention group claimed a higher degree of fullness (t(29) = − 6.717, *p* < 0.001) with a lower degree of hunger (t(29) = 4.156, *p* < 0.001), desire to eat (t(29) = 5.329, *p* < 0.001) and prospective food consumption (t(29) = 4.400, *p* < 0.001).

Table 9 compares the total calories and macronutrients compositions of dietary intake between the placebo and intervention groups. Respondents in the intervention group had a significantly lower calories (t(29) = 3.566, *p* = 0.001) and fat intake (t(29) = 2.197, *p* = 0.036) as well as higher dietary fiber intake (t(29) = − 12.843, *p* < 0.001) than the placebo group. Meanwhile, no significant difference was observed in carbohydrate and protein intakes (t(29) = − 1.936, *p* = 0.063 and t(29) = 1.309, *p* = 0.201, respectively).

**Table 9:** Total calories and macronutrients compositions of dietary intake (*n* = 30)

Dietary Variables	Placebo	Intervention	*p*-value
Energy intake (kcal)	1920 ± 259	1837 ± 269	0.001
Macronutrients			
Carbohydrate intake			0.063
Total (g)	224.2 ± 49.8	237.4 ± 46.4	
% from energy intake	46.1	47.3	
Dietary fiber (g)	6.3 ± 2.4	10.9 ± 1.6	< 0.001
Protein intake			0.201
Total (g)	78.0 ± 16.3	73.5 ± 13.1	
% from energy intake	16.3	16.0	
Fat intake			0.036
Total (g)	80.2 ± 17.8	74.9 ± 18.4	
% from energy intake	37.6	36.7	

The presented results are indicated as mean values ± standard deviation

Tested using paired t-test

## Discussion

### Glycemic response

The findings from this study revealed how the formulated MMT peel powder can lower postprandial blood glucose in adults at risk of T2DM. Results suggest some reduction in postprandial glucose peaks at certain time points in the intervention group compared with placebo group. These results are in accord with recent studies that indicating significant lower blood glucose levels observed in test meals containing dietary fiber [31–33]. However, a higher blood glucose levels at 120 min in the intervention group could be attributed to the presence of slowly digestible starch in the formulated MMT peel powder belonging to the intervention group which digested between 20 min and 180 min [34]. The study outcome was in line with a study that reported the postprandial blood glucose level was significantly higher for the intervention treatments (yellow mustard mucilage and flaxseed mucilage) when compared to the control treatment at 120 min [35]. Similar FPG concentration between the two treatment groups was in line with the findings of other studies [17, 35]. This finding can provide power to the study’s findings because it minimizes possible confounding factor of various FPG which may affect the overall postprandial glycemic response end points [35]. The FPG is minimally affected by the amount or rate of glucose absorption of the previous meal and reflect the glucose production rate in the liver (the two important processes are glycogenolysis and gluconeogenesis) [36].

iAUC describes the glycemic response to food more accurately [37]. It is a suitable indicator of glycation potential [38]. In this study, the iAUC indicates the changes in the blood glucose levels over 2 hr. after study breakfast consumption, in which a significantly lower value was observed in the intervention group. This finding mirrored one of the previous study that has examined a significantly lower iAUC after ingestion of the intervention treatment meals [39]. Besides, the finding on the significantly lower blood glucose CMax in the intervention group is consistent with the previous study which reported significant lower blood glucose Cmax for intervention treatments than the control treatment [35]. Previous investigations reported a difference of > 16% [40] or 20% [41] in blood glucose iAUC which was considered as clinically and physiologically relevant. This suggested that formulated MMT peel powder had the potential to lower glycemic response via modulation and attenuation of postprandial hyperglycemia which lead to improved blood glucose management [42] as evidenced by 26.8% reduction of blood glucose 2 hr. iAUC.

The suppression of acute glucose elevation after ingestion in this study could be attributed to the evidence that affirmed the significant role of dietary fiber in glucose metabolism of healthy people and those with impaired glucose metabolism [43, 44] by improving glycemic control [45–47] via various mechanisms. It depends on the physicochemical properties of soluble and insoluble dietary fiber in the gastrointestinal tract (GIT) [48]. The three most popular proposed mechanisms are namely: delayed gastric emptying, delayed amylolysis in the small intestine and delayed sugar absorption from the small intestine [49].

Soluble fiber is closely linked to the glycemic index concept which associated with satiety, short-term gut hormone, glucose and insulin responses [41, 50, 51] due to its viscosity and gel-forming properties [52, 53]. The water soluble dietary fiber forms a viscous solution or gel in the stomach that delay gastric emptying from the stomach to the small intestine and physically reduce the rate of starch digestion and absorption in the small intestine [53–55] by forming an effective unstirred layer in the intestinal wall [32]. Next, the changes in the solution viscosity influence glucose diffusion by exerting greater resistance and hence delay its transportation across the intestinal mucosa [56]. Besides, it diminishes the interaction and mixing of macronutrients with digestive enzymes and reduces intestinal permeability which delays and minimizes glucose absorption [57, 58]. A recent study also demonstrated that soluble fiber can suppress α-amylase activity in in vitro starch hydrolysis via non-competitive means [59]. Consequently, a lower postprandial blood glucose level was attained with subsequently reduced insulin secretion and glycated haemoglobin (HbA1c) levels [40].

Insoluble fiber can act as a physical barrier by keeping the starch trapped within the fiber network, causing starch integrity to deteriorate by lowering its diameter and modifying its shape [60]. Reduced starch diameter resulted in less surface area exposed to enzyme action, loweing starch digestibility [12]. A study reported that adding soy and amaranth to a biscuit reduced its rate of the starch digestion [61]. Dietary fiber can also reduce water availability for starch swelling and starch gelatinization attributed to its water holding capacity [12]. Besides, the insoluble fiber can trap the enzyme and minimize its access to starch [60]. This is supported by a research that reported insoluble fiber can exert α-amlyase inhibition effect in mixed ways [59]. As a result, starch is less vulnerable to enzymatic hydrolysis during human digestion which lead to slower rate of starch conversion to glucose [61].

Moreover, insoluble fiber suppress glucose diffusion through the intestinal barrier which delays glucose absorption with the subsequent lowered postprandial glycemic response [62]. Furthermore, insoluble fiber triggers an elevated release of glucose-dependent insulinotropic polypeptide which is an incretin hormone. It can stimulate the postprandial insulin liberation to promote glucose absorption into adipose, liver and skeletal muscle cells from the blood circulation [63]. Additionally, the postprandial blood glucose lowering effect could be attributed to the inhibitors in the MMT peel powder and its dietary fiber matrix such as polyphenols might interact with the enzyme’s active site and thus inhibit its activity [64].

The interaction and mutual effects of the gut microbiota and soluble fiber are also one of the mechanisms [65, 66]. Fermentation of soluble fiber raises microbiota diversity and short chain fatty acids (SCFAs) production [55, 65, 67]. SCFA especially butyric and propionic acids can facilitate gut peptides synthesis for instance glucagon-like peptide-1 and peptide YY which lead to improvement of insulin secretion [68] and HbA1c levels [69]. This is because SCFA can suppress glucose metabolism via the restriction of glucose transporter type 4 and compete with insulin-sensitive tissues [12]. However, the fermentation process requires a longer time in order to exert therapeutic effects, which is observed in an acute study that reported an insignificant positive effect on postprandial glycemia [70]. Hence, the mechanism and effectiveness of dietary fiber in reducing postprandial glycemia could be influenced by the experiment duration.

In this study, the acute reduction of postprandial 2-hr blood glucose was speculated to be attributed to the delayed carbohydrates absorption instead of colonic fermentation [71]. This is because colonic fermentation takes a longer time (6 hr) to occur by exhibiting its therapeutic hypoglycemic effect [72]. The ingredients such as dietary fiber, polyphenols, sweetener and other bioactive compounds present in the formulated MMT peel powder may interact synergistically to modulate glycemic response. However, the mechanisms of action for its blood glucose lowering effect warranted further investigation [17]. This is because the underlying mechanisms are complex and cannot be described exclusively by a single element [19]. Understanding the underlying mechanisms is crucial to help design foods that can reduce the glycemic response more effectively [49].

### Perceived satiety and food intake

AUC determination was preferred as a single satiety data at specific time points are not physiologically or statistically independent [73]. It was speculated that the taste and mouthfeel are probably the contributing factors to subjective feelings of fullness [74]. The taste serves as a nutrient sensor, stimulating the brain as well as the gut concerning the nutrients delivery via the cephalic phase response [75, 76]. The mastication of dietary fiber-rich foods require time and effort which prolongs oral exposure and provided time for signals that control satiety sensations [73]. Therefore, a greater oral and sensory exposure of viscous stimuli caused delayed gastric emptying with subsequent early meal termination [77] and increased satiety [76, 78].

Apart from that, the research also investigated the effect of food form in terms of viscosity regarding satiety. The thickening agents added to the food were dietary fibers which altered the viscosity [79]. The thick sensory cue was related to greater expectations of satiation [76, 80]. For instance, a greater viscosity of a semi-solid chocolate pudding lead to a slower eating rate, gastric response alteration and higher subjective satiety [81]. Also, the thickness and creaminess of a yogurt beverage altered the perception of satiation [82] and diminished subsequent lunch intake [83]. The evidence above best supported the findings of current study in which greater perceived satiety was observed in respondents who consumed more viscous formulated MMT peel powder. Next, a slower eating rate attributed to the presence of dietary fiber was also associated with a lower energy intake as reported by a systematic review [84].

Significantly lower energy and fat intake as well as higher fiber intake were also observed in the intervention group compared with the placebo group. Similar findings were reported in which higher dietary fiber intake was observed in the intervention group whereas no significant difference was observed in carbohydrate and protein intakes [85]. The higher dietary fiber intake in the intervention group was contributed by the high dietary fiber test food compared to other test food (control) which is low in dietary fiber. Appetite and energy intakes are dependent on the rigorous coordination of interrelated gastric and small intestine mechanisms, induced by the interaction of these organs with ingested nutrients [86]. Previous reviews had been reported on the type and amount of fibers needed to maximize satiety and control appetite and food consumption [87–89]. Typically, the fiber inclusion can reduce appetite in approximately 40% of the published studies as compared to the control [89, 90].

### Practical considerations

A daily consumption of 36 g of formulated MMT peel powder (5 g dietary fiber) accounted for 25% of the dietary fiber RNI (20 g). Hence, in order to meet the 20 g/d of dietary fiber as recommended, a total of 144 g of formulated MMT peel powder was needed. However, further research is warranted to investigate any side effects of the amount of consumption.

### Strengths

The present study has several significances. Firstly, it is the first study to date attempt to investigate the acute effect of consuming formulated MMT peel powder on glycemic response, perceived satiety and food intake in adults at risk of T2DM. This study also had another advantage of using the randomized crossover study design, which can eliminate the between-respondent variability. Since each of the respondent serves as his or her control, the influence of confounding covariates is minimized. Subsequently, this can lead to a reduced variability in the outcomes being examined and increase the precision of estimation. Furthermore, an equal amount of available carbohydrate was used in the intervention and placebo groups. This is because the higher amount of available carbohydrate may be contributed to the higher glycemic response. Therefore, the postprandial blood glucose lowering effect in this study can be attributed to the presence of dietary fiber in the intervention groups. In the existing literature, the serving amounts are not always proportioned to the amount of available carbohydrate. So, it is crucial to make sure the test meals contained similar available carbohydrate otherwise the focus could be on the nutritional variations [19].

### Limitations

Several limitations of this study are acknowledged. First of all, since this is an acute feeding study, thus the acute findings cannot translate to long-term benefit. Next, due to limitation in budgeting, the biochemical parameters such as serum insulin, lipid profiles and appetite hormones were not assessed. Therefore, satiety was only measured using subjective means instead of assessed by physiological markers. The therapeutic effect of formulated MMT peel powder could not be generalized to other populations, particularly those diagnosed with prediabetes or T2DM. Additionally, the sample was only drawn from adults at risk of T2DM in Kuala Nerus, Terengganu due to financial constraints and predominantly homogeneous Malays population. This does not reflect the ethnic diversity of Malaysian. Therefore, the findings could not be generalized to other Malaysian population of adults at risk of T2DM from other races such as Chinese and Indians in which their dietary intake are likely to be different.

## Conclusions

The findings of the present study indicate that short-term consumption of formulated MMT peel powder may improve glycemic response, increase perceived satiety and reduce food intake in adults at risk of T2DM, with the potential to be utilized as a functional beverage. A medium-to long-term clinical trial is warranted to determine whether taking this formulated MMT peel powder on a daily basis has an influence on health outcomes.

## Data Availability

The datasets generated and/or analyzed during the current study are not publicly available due to maintenance of the respondents’ privacy but are available from the corresponding author upon reasonable request.

## Abbreviations

ANOVA: Analysis of variance
AUC: Area under the curve
SCFAs: Short chain fatty acids
CMax: Maximum concentration
CONSORT: Consolidated standards of reporting trials
FPG: Fasting plasma glucose
FINDRISC: Finnish type 2 diabetes risk assessment tool
HbA1c: Glycated hemoglobin
hr.: Hour
iAUC: Incremental area under the curve
min: Minute
mL: Milliliters
mmol/L: Millimole/liter
MMT: Melon Manis Terengganu
n: Sample size
SD: Standard deviation
SOP: Standard operating procedure
T2DM: Type 2 diabetes mellitus
UHREC: UniSZA Human Research Ethics Committee
UniSZA: Universiti Sultan Zainal Abidin
WC: Waist circumference
